# Substance Use and Incidence of Psychosis Diagnoses Among Gay, Bisexual, and Other Men Who Have Sex with Men Compared with Males from the General Population: A Matched Cohort Study from Metro Vancouver, Canada

**DOI:** 10.1080/10826084.2026.2682299

**Published:** 2026-06-11

**Authors:** Aki Gormezano, Arlo Adams, Lu Wang, Jason Trigg, Michael Budu, Robert S. Hogg, Santiago Aguilera-Mijares, Mark Hull, Justin Barath, Paul Sereda, Allan Lal, Joseph Cox, Trevor A. Hart, Daniel Grace, Darrell H.S. Tan, Nathan J. Lachowsky, David M. Moore

**Affiliations:** a British Columbia Centre for Excellence in HIV/AIDS, Vancouver, BC, Canada; b School of Health Sciences, Faculty of Human and Health Sciences, University of Northern British Columbia, Prince George, BC, Canada; c School of Population and Public Health, University of British Columbia, Vancouver, BC, Canada; d Faculty of Health Sciences, Simon Fraser University, Burnaby, BC, Canada; e Research Institute of the McGill University Health Centre, Montréal, QC, Canada; f Department of Epidemiology, Biostatistics and Occupational Health, School of Population and Global Health, McGill University, 2001 McGill College, Montréal, QC, Canada; g Department of Psychology, Faculty of Arts, Toronto Metropolitan University, Toronto, ON, Canada; h Dalla Lana School of Public Health, University of Toronto, Toronto, ON, Canada; i Department of Medicine, Temerty Faculty of Medicine, University of Toronto, Toronto, ON, Canada; j St. Michael’s Hospital, Unity Health Toronto, Toronto, ON, Canada

**Keywords:** Psychosis, substance use, sexual minority, administrative health data, health disparities

## Abstract

**Background:**

Gay, bisexual, and other men who have sex with men (GBM) report higher use of psychoactive substances, which may contribute to many adverse outcomes.

**Method:**

We examined the incidence of psychosis and associated substance use among GBM compared with males from the general male population. We analyzed data from 798 sexually active GBM from two cohort studies based in Vancouver, Canada, linking their survey data to administrative health records. We matched GBM (1:5) by age and geography to HIV-negative males. We determined incident psychosis using ICD-9/10 codes and examined associated factors using Poisson regression.

**Results:**

Incidence was 1.74 per 100 person-years for GBM and 0.64 for their matches (risk-ratio = 2.71; 95% CI:1.95–3.78). Among GBM, opioid use, living with HIV, unstable housing, and anxiety symptoms were independently associated with psychosis.

**Conclusions:**

Addressing social determinants of health for GBM such as unstable housing, particularly among those living with HIV, may reduce the burden of substance-use associated psychosis.

## Introduction

Psychosis is a complex mental health condition characterized by delusions and/or hallucinations without insight into their pathologic nature (American Psychiatric Association DSM-5 Task Force, 2013; WHO, 2019). Rather than presenting as a stable or uniform condition, psychosis is commonly experienced as dynamic and heterogeneous, involving fluctuations over time and periods of acute distress, altered perception, and significant disruptions to identity, autonomy, and everyday functioning, even beyond acute episodes (Fusar-Poli et al., 2022). Estimates of its prevalence vary widely by study design and case-finding setting; however, a large systematic review in the general population reported median point and lifetime prevalence estimates of 3.89 and 7.49 per 1,000 persons, respectively (Moreno-Küstner et al., 2018).

Psychosis is influenced by a multitude of factors, including substance use-related determinants. A substantial body of evidence links psychoactive substance use—particularly stimulants such as amphetamines—to increased risk of psychosis in the general population (McKetin et al., 2019). Substance-related psychosis may arise through multiple pathways, including acute intoxication effects, prolonged high-frequency use, and withdrawal-related processes, with risk varying by substance type and pattern of use (Lozano-López et al., 2021; McKetin et al., 2019; Moreno-Gámez et al., 2022).

In parallel, sexual minorities overall show higher rates of psychotic experiences and disorders than heterosexuals (Selten et al., 2024). These disparities are thought to reflect the cumulative effects of social adversity, including stigma, discrimination, violence exposure, and barriers to care, which may contribute to chronic stress and increased vulnerability to adverse mental health outcomes (Selten et al., 2024; Tsuyuki et al., 2017). Structural factors such as housing instability have also been consistently linked to psychosis risk (Ayano et al., 2019).

Gay, bisexual, and other men who have sex with men (GBM) use psychoactive substances such as amphetamines and alcohol at higher rates than their heterosexual counterparts (Lea et al., 2013; Schmidt et al., 2016). Sexualized substance use, including “chemsex” and “party-n-play,” and often involving crystal methamphetamine and GHB/GBL^1^, reflect unique socio-sexual cultures among GBM, particularly in urban centers and among those living with HIV (Bourne & Weatherburn, 2017; Rich et al., 2016). Beyond its association with sexual behaviors linked to HIV and other sexually transmitted infection (STI) transmission (Hart et al., 2023, 2024), elevated psychoactive substance use among GBM may have mental health consequences, including psychosis (McKetin et al., 2019).

A systematic review focused on GBM engaged in sexualized substance use showed rates of psychosis symptoms and diagnoses ranging from 6.7% to 37.2% (Moreno-Gámez et al., 2022). This wide range likely reflects differences in study populations, definitions of psychosis, and methods of ascertainment, but nonetheless points to a potentially elevated burden in this population. Complementing these findings, recent work among GBM living with HIV has shown that psychotropic substance use—particularly methamphetamine—was associated with substantially higher odds of psychotic disorders compared with those not using these substances, with the strongest associations observed among individuals reporting more intensive or frequent methamphetamine use (Lee et al., 2023). Despite these findings, no studies to date have examined the incidence of psychosis specifically among GBM or assessed how specific types of psychoactive substance use are associated with new-onset psychosis.

We compared incident psychosis diagnoses between GBM identified through two cohort studies in Metro Vancouver, Canada with matches from the general male population using administrative health data (Objective 1). We then used behavioral data from the GBM-specific observational cohort studies to examine factors associated with incident psychosis diagnoses with a nuanced focus on different types of substance use (Objective 2).

## Materials and methods

### Momentum I & II study design and participants

We used data from two prospective observational cohort studies in Metro Vancouver: Momentum I (2012–2019; Moore et al., 2016) and Momentum II (2017–2023; Hart et al., 2021). For both studies, we recruited sexually active GBM who were ≥16 years of age. Other inclusion criteria included self-identifying as a cisgender or transgender man, being able to read English, currently living in Metro Vancouver, and reporting sex with another man in the previous six months (P6M). We used Respondent-Driven Sampling (RDS) to recruit participants for both cohort studies, with seeds drawn from community contacts who could recruit up to six peers with small incentives for each completed referral (Lachowsky et al., 2016; Sang et al., 2023; Yauck et al., 2021). RDS sampling has been shown to be an effective and valid method for recruiting study participants from marginalized populations who do not have a comprehensive sampling frame (Heckathorn & Cameron, 2017).

Participants completed computer-assisted self-interviews and a nurse-led screening for HIV and STIs at baseline and at return study visits every six to 12 months. We provided participants with an honorarium of $50 CAD per visit. Self-interview questions included demographics, sexual risk behaviors, and substance use within the P6M. Notably, this did not include questions about past psychosis diagnoses. We separately asked participants to consent to linking their study data with administrative health records in British Columbia (BC). In Momentum I, we initially sought this consent only from participants living with HIV at enrollment. After a January 2015 protocol amendment, we also requested consent for data linkages from HIV-negative participants. We asked all Momentum II participants to consent to link at enrollment. Deterministic linkages to the administrative data were based on a combination of at least two of the following: personal health number, legal name, and/or date of birth.

### COAST data linkage and outcome measures

We measured incidence rates of psychosis through administrative health data as part of the COAST (Comparison of Outcomes and Service Utilization Trends) study.^2^ The aim of the COAST study is to determine health outcomes for adults (≥ 19 years of age) living with HIV in BC by comparing them to a random selection of HIV-negative population controls (Budu et al., 2025; Eyawo et al., 2018). GBM participants from the Momentum studies were matched with HIV-negative COAST participants whose administrative health records listed their sex as male in a 1:5 ratio based on age and Local Health Area (LHA), a geographical unit of healthcare service delivery.^3^ Matching was limited to age and residential region because these variables were available in both the Momentum and COAST datasets and represent key structural determinants of health and healthcare access. Additional sociodemographic characteristics—such as education, employment, and housing—were not available in the COAST administrative data and therefore could not be incorporated into the matching process or used for direct comparison between groups. We did not match participants on the basis of HIV status, as most men living with HIV in Metro Vancouver acquire HIV through having sex with other men, and our primary interest was in comparing GBM with non-GBM (BC Centre for Excellence in HIV/AIDS, 2024).

COAST contains information from physician billing data through the Medical Services Plan (MSP) (BC Ministry of Health, 2020) and hospitalizations through the Discharge Abstract Database (DAD) (Canadian Institute of Health Information, 2020). We defined psychosis as the occurrence of one hospitalization or one physician visit coded with applicable diagnostic codes. Specifically, we selected diagnostic codes from the psychosis-related sections of the World Health Organization’s ICD-9 and ICD-10 classification systems, including ICD-9 psychosis codes (291, 292, 295, 297, 298) and select diagnoses from the ICD-10 psychotic disorders block (F20–F29; World Health Organization, 1977, 1992). We detail all diagnostic codes and corresponding data sources in Table 1. All participants included in the analysis had at least one year of administrative follow-up data until March 2020.

### Explanatory measures

For Objective 1, the variables we included in analyses from the COAST data were HIV status, age, postal code, and residential region (derived from health authority and grouped into “City of Vancouver,” “Suburban Vancouver” (i.e., non-Vancouver Lower Mainland), and “other regions”).

For Objective 2, key variables we included from the Momentum studies are detailed below:

- Sociodemographic data: We assessed age, annual personal income, ethnoracial group (white, Asian, Other), sexual orientation (Gay, Bisexual, Other), gender identity (cisgender men, trans and nonbinary men), education level, current employment, current access to a regular primary healthcare provider (yes, no), and housing status (stable, unstable).
- HIV status: We grouped self-reported HIV status as negative/unknown and living with HIV.
- Anxiety and depression: We used separate sub-scores for anxiety and depression from the Hospital Anxiety and Depression Scale (HADS; Zigmond & Snaith, 1983). The HADS assesses symptoms suggestive of anxiety and depressive disorders rather than providing a clinical diagnosis. Following established guidelines and prior studies of GBM (e.g., Zahran et al., 2024), we dichotomized each subscale into “normal/mild” (scores < 11) and “moderate/severe” (scores 11–21), with higher scores indicating greater symptom severity and increasing likelihood of clinically significant affective symptoms.
- Sexual behavior:

- Any condomless anal sex with a serodifferent partner in the past 6 months (P6M) (Yes/No).
- Any sex exchanged for drugs/money/other goods in the P6M (Yes/No).

Substance Use:

- We used ASSIST (The Alcohol, Smoking and Substance Involvement Screening Test) to comprehensively assess any substance use in the P6M (World Health Organization ASSIST Working Group, 2002). We used P6M ASSIST substance use groups for cannabis, cocaine, amphetamines, sedatives, hallucinogens, and opioids in analyses. The amphetamines ASSIST group included ecstasy/MDMA, mephedrone, speed, methylphenidate, amphetamine-dextroamphetamine, and crystal methamphetamine (which we also assessed individually).
- In addition to ASSIST groups, we included variables for any P6M use of “sex drugs” (crystal meth, GHB, erectile drugs) and “club drugs” (ecstasy, mushrooms, cocaine; Card, Armstrong et al., 2018).
- Self-report of ever having received a substance use disorder (SUD) diagnosis from a physician, psychologist, or psychiatrist (Yes/No).
- Injection drug use in the P6M (Yes/No)

### Ethics

Ethics approval for these studies was granted by the research ethics boards at the University of British Columbia, the University of Victoria, and Simon Fraser University (H11–00691, H16–01226, and H22–02875). All participants provided written informed consent to the study procedures.

### Statistical analyses

Population Data BC linked all GBM from Momentum who consented to the data linkage to the COAST database, then extracted data for all successfully linked participants. Momentum participants were then matched on age (+/−1 year) and LHA with HIV-negative males from COAST’s random sample of BC residents in a 1:5 ratio. To assess the matching results, we compared the proportions across age groups and residential regions for Momentum GBM participants and their matches using chi-square tests.

We estimated psychosis incidence after excluding individuals with evidence of prior psychosis. Prevalent cases were identified using a three-year lookback window anchored to each Momentum participant’s baseline date. For Momentum GBM participants, this window covered the three years preceding their Momentum I or II enrollment; for their COAST matches, the same baseline date of the corresponding Momentum participant was used to define the three-year lookback period. Individuals who met the psychosis case definition in COAST data during the relevant lookback period were excluded from the incidence cohort. We then calculated psychosis incidence rates for GBM and their matches, as well as the incidence rate ratio (IRR) comparing the two groups (Objective 1). To assess the potential influence of HIV status, we repeated these incidence estimations after excluding Momentum GBM living with HIV and their matches (sensitivity analysis).

We then identified factors associated with incident psychosis diagnoses among GBM using data collected during the Momentum studies (Objective 2). We conducted univariable and multivariable models using Poisson regression with generalized estimating equations (GEE) with an autoregressive correlation structure (Ballinger, 2004; Hanley et al., 2003; Zorn, 2001). Due to the limited number of incident psychosis events, we identified a pool of candidate variables for entry into the multivariable model to support model stability based on theoretical considerations, research interest (including clinical relevance), and univariable *p*-values below our established alpha of 5%. This subset was defined prior to model optimization, and not all variables with statistically significant univariable associations were eligible for inclusion in the multivariable model due to constraints related to the limited number of incident psychosis events. As an exploratory analysis to account for polysubstance-use (Card, Lachowsky et al., 2018), we also examined two-way interactions between amphetamines, opioids, and sedatives. We assessed interactions between these ASSIST categories specifically because they were significant at the univariable level and represented substance use combinations with theoretical relevance and sufficient prevalence for stable estimation. We also examined a model replacing opioid use with amphetamines to assess the robustness of substance-specific associations. Our final multivariable model included the candidate variables that minimized the Quasi-likelihood Information Criterion (QIC) and assessment of type III tests of fixed effects (Pan, 2001; Wang, 2014). Variables identified as “Not selected” were those that were initially entered into the multivariable model and subsequently removed on this basis. Variables without the “Not selected” designation were never entered into the multivariable model because they were not selected as candidate variables. We conducted all analyses using SAS 9.4 (SAS Institute Inc., 2023).

## Results

Of 774 participants in the Momentum I study, 495 (64.0%) consented to the COAST data linkage. However, 85 participants lacked the requisite three years of pre-enrollment administrative data and five participants could not be linked to the administratve data, leaving 405 participants from Momentum I. For Momentum II, 813 of 822 participants (99%) consented to COAST data linkage. This group included 149 participants who had also taken part in Momentum I; these individuals were only counted once when creating the combined sample. Among the remaining Momentum II participants, 35 could not be linked to the administrative data, and 128 did not meet the three-year pre-enrollment data requirement, leaving 650 participants from Momentum II. After removing 108 participants from the Momentum I and II cohorts that had less than one year of follow-up data in COAST, the final analytic sample included 798 unique GBM participants from the Momentum cohorts.

Supplemental Table S1 presents comparisons between the the 344 excluded GBM and the analytic sample. Excluded GBM were significantly younger, more ethnoracially diverse, and less likely to be living with HIV, to have exchanged sex for drugs/money/goods in the P6M, to have been diagnosed by a doctor with a substance use disorder, or to report P6M opioid or amphetamine use.

The 798 Momentum GBM retained in the analytic sample were matched to 3,990 HIV-negative males from the COAST cohort. Comparisons between Momentum participants (median age = 35.0 years) and their matches (median age = 35.0 years) showed no significant differences by age or residential region (Table 2). Additional sociodemographic characteristics were not available for COAST matches and therefore could not be compared between groups.

In Supplementary Table S2, we present the frequency with which each ICD-9 and ICD-10 diagnostic code contributing to the psychosis case definition was recorded in the administrative data. Across Momentum GBM and COAST matches, the majority of identified cases were captured through diagnostic codes with high specificity for primary psychotic disorders—particularly ICD-9 codes 292 (drug-induced psychoses), 295 (schizophrenic disorders), and 298 (other nonorganic psychoses), and ICD-10 F20 (schizophrenia). Codes representing transient or secondary presentations (e.g., brief psychotic disorder, F23; persistent delusional disorders, F22) were comparatively rare.

In Table 3, we describe the demographic characteristics and self-reported substance use behaviors of Momentum GBM reported at enrollment. Most participants were white/Canadian/European (73%), gay (88%), cisgender (96%), educated above high school (83%), currently employed (72%), and had a regular primary care provider (77.2%). A minority of participants were living with HIV (26%) and unstably housed (9%). P6M substance use was highest for cannabis (60%) followed by amphetamines (37%). Fifteen percent of participants self-reported ever having a doctor-diagnosed SUD. A quarter (25%) of participants had HADS scores indicative of moderate to severe symptoms of anxiety, and 5% of participants had scores indicative of moderate to severe symptoms of depression.

In Table 4, we present the incidence of psychosis diagnoses for Momentum GBM and their matches. At baseline, 7.1% (*n* = 57) of GBM had psychosis diagnoses compared with 3.4% (*n* = 137) of matches. The incidence of psychosis was 1.74 (95% CI 1.33, 2.28) per 100 person-years among GBM and 0.64 per 100 person-years (0.53, 0.78) among the matches for an IRR of 2.71 (1.95, 3.78).

As a sensitivity analysis, we also assessed the incidence of psychosis by excluding all participants living with HIV from Momentum (*n* = 217) and their COAST matches (*n* = 1085) (Table 5). At baseline, 4.8% (*n* = 28) of GBM had psychosis diagnoses compared to 3.0% (*n* = 88) of matches. The incident psychosis diagnosis rate with the participants living with HIV removed was 1.03 (95% CI 0.68, 1.56) among GBM and 0.45 (95% CI 0.34,0.60) among matches for an IRR of 2.27 (95% CI 1.38, 3.74).

Using univariable and multivariable Poisson regression with GEE, we assessed the association of the variables described in Table 3 with psychosis diagnoses identified in the administrative health data (Table 6). In univariable analyses, psychosis diagnoses were associated with being unemployed (IRR = 0.46, 95% CI [0.26, 0.78]), having a regular primary healthcare provider (IRR = 3.99, [1.24, 12.77]), having unstable housing (IRR = 0.21, [0.11, 0.39]), living with HIV (IRR = 3.35, [CI 1.93, 5.80]), having moderate to severe anxiety (IRR = 3.97, [2.25, 7.00]), having moderate to severe depression (IRR = 3.81, [1.80, 8.07]), and having exchanged sex for drugs/money/other goods in the P6M (IRR = 4.26, [2.25, 8.07]). With univariable analyses for substance use specifically, psychosis diagnoses were associated with P6M injection drug use (IRR = 4.90, [2.68, 8.96]), self-reported lifetime doctor-diagnosed SUD (IRR = 2.36, [1.29, 4.32]), P6M use of sex drugs (IRR = 1.92, [1.12, 3.30]), P6M amphetamine use (including crystal meth) (IRR = 2.17, [1.27, 3.72]) as well as P6M crystal meth use specifically (IRR = 4.57, [2.62, 7.96]), P6M sedative use (IRR = 2.16, [1.21, 3.86]), and P6M opioid use (IRR = 4.21, [2.14, 8.27]). Notably, the univariable association between psychosis diagnoses and P6M cannabis use was not significant.

In the multivariable model, only unstable housing (adjusted IRR [aIRR] = 0.40, 95% CI [0.20, 0.82]), living with HIV (aIRR = 3.29, [1.72, 6.32]), moderate to severe anxiety scores (aIRR = 3.98, [2.17, 7.32]), and P6M opioid use (aIRR 2.38, [1.09, 5.20]) were retained. As an exploratory analysis, we assessed the two-way interactions between P6M use of amphetamines, opioids, and sedatives; none of these were statistically significant (data not shown). We also examined a model replacing opioid use with amphetamines, which was also not statistically significant (data not shown).

## Discussion

Compared with HIV-negative males from the general population of Metro Vancouver, our matched cohort of GBM had a higher incident rate of psychosis diagnoses. One possibility was that the higher incidence rate of psychosis among GBM compared with their matches was due to men in the GBM sample living with HIV, which is a known risk factor for psychosis through a combination of organic mechanisms (e.g., neuroinflammation, CNS opportunistic infections, ART neurotoxicity) and intersecting social determinants such as stigma, violence exposure, and structural marginalization (Helleberg et al., 2015; Marinho et al., 2016; Tsuyuki et al., 2017). However, our sensitivity analysis showed that HIV-negative GBM still had a significantly higher incidence rate of psychosis than matched general population males, ruling out the possibility that the difference in incident psychosis we found was due to differences in HIV status alone. Further, our multivariable model for Momentum GBM showed significant associations between incidence of psychosis and opioid use, anxiety, and unstable housing *after* controlling for HIV status.

There are a few past studies that have assessed the incidence of psychosis among GBM, but these had comparatively small samples (*N* < 300), were cross-sectional, and mostly used self-report (Bolton & Sareen, 2011; Lu et al., 2023; Post & Veling, 2021). The present study provides the most comprehensive assessment of psychosis among GBM to date, estimating incidence based on longitudinal administrative health data from 798 participants. Our outcome measure was based on diagnoses from clinicians found in administrative health data, and we were able to include a matched comparison from the general male population. Consistent with past studies, we found that living with HIV (Helleberg et al., 2015; Marinho et al., 2016), having unstable housing (Ayano et al., 2019), and anxiety (Fusar-Poli et al., 2012; Michail & Birchwood, 2009; Morales-Muñoz et al., 2022) were positively associated with psychosis.

Our findings for substance use and psychosis among GBM were only partially consistent with past studies. At the univariable level, we found that psychosis was positively associated with amphetamine use, sedative use, crystal methamphetamine use, and opioid use. However, when examining independent associations *via* a multivariable model, only opioid use was retained in the final model. In an exploratory analysis replacing opioid use with amphetamines, this association was not statistically significant. Exploratory analyses examining interactions between substance use types (including amphetamines) were also not statistically significant. Taken together, these findings suggest that the lack of an independent association for amphetamines is not simply a consequence of model specification. This finding is surprising given that psychosis has been previously shown to be strongly associated with amphetamine (McKetin et al., 2019) and cannabis use (Marconi et al., 2016) in past studies of the general population. Further, the limited past work on the association between opioid use and psychosis shows mixed results (Habiger et al., 2016; Siakabanze et al., 2025). One possible explanation of our current findings is that the association between psychosis and opioid use reflects fluctuating use or withdrawal, rather than consistent use, given case reports of psychotic symptoms following opioid withdrawal (e.g., after stopping buprenorphine; Lozano-López et al., 2021; Maremmani et al., 2014). An additional, non–mutually exclusive interpretation is that opioid use may function as an attempt to cope with or self-regulate distress during earlier, prodromal stages of psychosis. Emerging psychosis is often preceded by anxiety, sleep disturbance, affective dysregulation, and nonspecific perceptual changes—symptoms for which opioids may provide transient relief (Fusar-Poli et al., 2022; Larson et al., 2010). This should be assessed in future research.

These findings should also be considered within the broader context of mental health disparities among GBM. Sexual minority populations are disproportionately exposed to social and structural stressors—including stigma, discrimination, violence, and barriers to care—which may contribute to chronic stress and increased vulnerability to adverse mental health outcomes (Selten et al., 2024; Tsuyuki et al., 2017). In this context, anxiety may represent both a marker of cumulative psychosocial stress and a potential pathway through which these exposures influence psychosis risk. The strong association we observed between anxiety and incident psychosis is consistent with evidence that affective dysregulation and anxiety symptoms often precede the onset of psychotic disorders and may reflect early or prodromal processes (Fusar-Poli et al., 2012; Michail & Birchwood, 2009; Morales-Muñoz et al., 2022). These dynamics may intersect with other factors identified in our analyses, including unstable housing and substance use, which can further exacerbate stress and limit access to supportive resources. For example, housing instability has been consistently linked to elevated risk of psychosis (Ayano et al., 2019), while substance use—particularly in the context of sexualized use among GBM—may both reflect and amplify underlying psychosocial vulnerabilities (Bourne & Weatherburn, 2017; Tsuyuki et al., 2017), contributing to elevated risk of psychosis in this population.

From a clinical and public-health perspective, our findings highlight opportunities for prevention of psychosis among GBM, particularly within services that already support GBM living with HIV and those engaged in substance use. Given the association we observed between psychosis and unstable housing, Housing First approaches such as the Canadian At Home/Chez Soi program—which provide immediate, non-contingent housing with embedded mental-health supports—may play a preventive role by reducing psychosocial stressors linked to psychosis onset or recurrence among people with severe mental illness (Aubry et al., 2015; Patterson et al., 2013; Stergiopoulos, Gozdzik et al., 2015; Stergiopoulos, Hwang et al., 2015; Stergiopoulos et al., 2019). Integrating GBM-affirming Housing First models with tailored addiction care and HIV services may help mitigate the compounded burdens of substance use, housing instability, and living with HIV that contribute to psychosis risk in this population, thereby reducing the likelihood of psychotic episodes across the life course.

## Limitations

Notably, administrative health data do not include information on participant sexual orientation. Given that an estimated 3% to 4.3% of men from the general population are expected to be GBM (Rich et al., 2018; Sorge et al., 2023), it is likely that some GBM were included in the general population comparison group from COAST. This misclassification would be expected to bias comparisons between Momentum GBM and their matches toward the null. To strengthen this comparison, we reduced the number of GBM in our COAST sample by only including men who were HIV-negative, as most new HIV infections among men in Vancouver are among GBM (BC Ministry of Health, 2020).

Our matching strategy was limited to age and residential region due to data availability in the COAST administrative dataset, which does not include key sociodemographic variables such as education, employment, or housing. As a result, we were unable to assess balance between groups on these characteristics or incorporate them into the matching process. Given that these factors are associated with psychosis risk, residual confounding may partially contribute to the observed differences in incidence between GBM and matched controls. Matching on age and geography nonetheless supports comparability on two important structural determinants of health and healthcare access, and the use of identical outcome ascertainment across groups reduces the likelihood of differential misclassification.

Our psychosis case definition relied on administrative diagnostic codes, which vary in clinical specificity and do not capture symptom severity or duration. However, as shown in Supplementary Table S2, most identified cases were ascertained using codes with high specificity for psychotic disorders (e.g., ICD-9 codes 292, 295, and 298; ICD-10 F20), while codes reflecting more transient or secondary presentations were comparatively rare. We also defined incident psychosis based on one hospitalization or one physician billing rather than two physician billings within a year, which may have increased sensitivity at the expense of specificity; applying a more restrictive two-billing definition would have yielded fewer events. Consistent with this, the baseline prevalence of psychosis we observed among matched males from the general population was higher than point-prevalence estimates reported in the literature, which could reflect our use of a one-billing case definition and the inclusion of diagnostic codes with varying levels of clinical specificity (Moreno-Küstner et al., 2018; Ochoa et al., 2012; Vassos et al., 2012). However, because identical case-definition criteria were applied to Momentum GBM and COAST matches, any misclassification of psychosis is likely to be non-differential.

Administrative data are also only moderately reliable compared with gold standard measures such as structured diagnostic interviews, although one systematic review suggested that >80% of administrative psychotic diagnoses using administrative data correspond with gold standard measurement (Davis et al., 2016). In addition, psychotic experiences are often dynamic and heterogeneous, with fluctuating insight and distress that may not consistently correspond to formal diagnostic thresholds (Fusar-Poli et al., 2022). Consequently, administrative diagnoses may incompletely reflect the experiential complexity and timing of psychosis as lived by participants.

In addition, we recruited a more representative sample of GBM through RDS, but excluded some of these men because we were unable to link them to their administrative psychosis data. As such, our sample of GBM was diverse but not necessarily representative of Metro Vancouver GBM. Further, given that excluded GBM were significantly less likely to be living with HIV or to report P6M amphetamine and opioid use—factors associated with psychosis in our analyses—the analytic sample may overrepresent individuals at higher underlying pyschosis risk. As such, incidence estimates may be somewhat elevated relative to the full cohort.

Finally, substance use among Momentum GBM was derived from ASSIST items and operationalized as binary P6M use (any vs none). Although the ASSIST permits more granular classifications (e.g., frequency- or risk-based categories reflecting problematic patterns of use), we adopted a binary approach to preserve statistical power and model stability given the limited number of psychosis events. As such, we were unable to examine potential dose–response relationships or distinguish between patterns of more intensive or problematic use.

## Conclusions

Our results provide longitudinal evidence that GBM appear to have an elevated risk of psychosis compared with HIV-negative males from the general population. The elevated risk of psychosis among GBM is related to living with HIV, opioid use, anxiety, and unstable housing, underscoring the importance of considering these intersecting factors preventing psychosis for this population.

## Supplementary Material

Supp 1

Supplemental data for this article can be accessed online at https://doi.org/10.1080/10826084.2026.2682299.

## Figures and Tables

**Table 1.** ICD-9 And ICD-10 diagnostic codes used to define psychosis in administrative health data.

Codes	Diagnostic description

***ICD-9 codes for physician visits (MSP)***	
291	Alcohol-induced psychoses
292	Drug-induced psychoses
295	Schizophrenic disorders
297	Delusional disorders
298	Other nonorganic psychoses
***ICD-10 codes for hospitalizations (DAD)***	
F20	Schizophrenia
F22	Persistent delusional disorders
F23	Brief psychotic disorder
F28	Other psychotic disorder not due to a substance or known physiological condition
F29	Unspecified nonorganic psychosis

*Note*. This table lists the ICD-9 and ICD-10 diagnostic codes used to identify psychosis in administrative health data, defined as one hospitalization or one physician visit. Codes were drawn from psychosis-related sections of the WHO ICD-9 and ICD-10 systems.

**Table 2.** Comparison of age and residential region of momentum GBM and HIV-negative male COAST matches.

Variables	Momentum (*N* = 798)	HIV-Negative COAST Matches (*N* = 3,990)	*p*-value

**Age** (in years)			0.978
Less than 30	245 (30.7)	1,210 (30.3)	
30 to 44	295 (37.0)	1,485 (37.2)	
45 years or older	258 (32.3)	1,295 (32.5)	
**Residential Region**			0.904
City of Vancouver	587 (73.5)	2,965 (74.3)	
Suburban Vancouver	204 (25.6)	990 (24.8)	
Other regions	7 (0.9)	35 (0.9)	

*Note*. COAST: Comparative Outcomes and Service Utilization Trends study. Values presented as n (%). *p*-values reflect differences between Momentum participants and HIV-negative COAST matches using chi-squared tests. Significance threshold set at *p* < 0.05.

**Table 3.** Baseline descriptives of momentum participants (*N* = 798).

Variables	*N*	*n*	(%)

**Age (categorical)**	798		
Less than 30		245	(30.7)
30 to 44		295	(37.0)
45 or more		258	(32.3)
**Ethnicity**	798		
White/Canadian/European		583	(73.1)
Asian		116	(14.5)
Other		99	(12.4)
**Sexual orientation**	798		
Gay		698	(87.5)
Bisexual		47	(5.9)
Other		53	(6.6)
**Gender identity**	798		
Cisgender man		768	(96.2)
Trans man and/or nonbinary		30	(3.8)
**Highest level of education**	798		
High school or less		136	(17.0)
Greater than high school		662	(83.0)
**Currently employed**	798		
No		222	(27.8)
Yes		576	(72.2)
**Currently have a regular primary health care provider**	797		
No		182	(22.8)
Yes		615	(77.2)
**Unstable housing**	790		
No		722	(91.4)
Yes		68	(8.6)
**Self-reported HIV status**	798		
HIV negative/unknown		587	(73.6)
Living with HIV		211	(26.4)
**HADS Anxiety Sub-Score**	787		
Normal/mild		588	(74.7)
Moderate/severe		199	(25.3)
**HADS depression sub-score**	786		
Normal/mild		744	(94.7)
Moderate/severe		42	(5.3)
**P6M Condomless anal sex with serodifferent partner**	793		
Yes		372	(46.9)
No		421	(53.1)
**P6M exchanged sex for drugs/money/goods**	794		
No		719	(90.6)
Yes		75	(9.4)
**P6M injection drug use**	798		
No		730	(91.5)
Yes		68	(8.5)
**Self-reported doctor-diagnosed substance use disorder diagnosis (ever)**	778		
No		660	(84.8)
Yes		118	(15.2)
**P6M substance use**			
Cannabis	796	480	(60.3)
Cocaine	797	231	(29.0)
Amphetamines	796	292	(36.7)
Crystal meth	795	140	(17.6)
Sedatives	795	191	(24.0)
Hallucinogens	795	181	(22.8)
Opioids	795	74	(9.3)

*Note*. P6M refers to “past 6 months.”

**Table 4.** Incidence of psychosis diagnoses among momentum GBM (*N* = 798) and HIV-negative male COAST matches (*N* = 3,990).

	n	Person years (PYRs)		Incidence rate per 100 PYRs (95% CI)	Incidence rate ratio (95% CI)
		Total	Median (Q1-Q3)		

**COAST matches**					
None	3,750	15,801.4	2.97 (1.87, 6.77)		
Incident psychosis	103	279.1	2.28 (0.75, 4.61)	0.64 (0.53, 0.78)	Ref.
Baseline prevalence	137 (3.4%)				
**Momentum participants**					
None	688	2,899.9	2.96 (1.90, 6.78)		
Incident psychosis	53	148.0	2.42 (0.90, 4.64)	1.74 (1.33, 2.28)	2.71 (1.95, 3.78)
Baseline prevalence	57 (7.1%)				

*Note*. COAST: Comparative Outcomes and Service Utilization Trends study; Q1: first quartile; Q3: third quartile; CI: confidence interval; Ref.: reference.

**Table 5.** Incidence of psychosis diagnoses among HIV-negative Momentum GBM (*N* = 581) and HIV-negative male COAST matches (*N* = 2,905).

	n	Person years (PYRs)		Incidence rate per 100 PYRs (95% CI)	Incidence rate ratio (95% CI)
		Total	Median (Q1-Q3)		

**COAST matches**					
None	2,767	10,898.5	2.57 (1.82, 6.70)		
Incident psychosis	50	132.4	2.12 (0.55, 4.74)	0.45 (0.34, 0.60)	Ref
Baseline prevalence	88 (3.0%)				
**HIV-negative momentum participants**					
None	531	2,070.3	2.52 (1.88, 6.69)		
Incident psychosis	22	67.9	2.27 (0.55, 5.55)	1.03 (0.68, 1.56)	2.27 (1.38, 3.74)
Baseline prevalence	28 (4.8%)				

*Note*. COAST: Comparative Outcomes and Service Utilization Trends study; Q1: first quartile; Q3: third quartile; CI : confidence interval; Ref.: reference.

**Table 6.** Univariable and multivariable generalized estimating equations for incident psychosis diagnoses among Momentum GBM (*N* = 741).

	Last visit*				All visits					
		No (*N* = 688)	Yes (*N* = 53)		Univariable models			Multivariable models		
Variables	N	n (%)	n (%)	*p*	IRR	(95% CI)	*p*	aIRR	(95% CI)	*p*

**Age (in years)**	741									
Less than 30		1 52 (22.1)	10 (18.9)	**0.013**	Ref.					
30 to 44		295 (42.9)	14 (26.4)		0.81	(0.36, 1.81)	0.604			
45 or more		241 (35.0)	29 (54.7)		1.62	(0.79, 3.31)	0.189			
**Annual personal income (CAD)**	739									
Less than $30,000		228 (33.2)	34 (64.2)	**<0.001**	Ref.					
$30,000 to $59,999		234 (34.1)	14 (26.4)		0.54	(0.29, 1.00)	**0.050**			
60,000 or higher		224 (32.7)	5 (9.4)		0.25	(0.10, 0.64)	**0.004**			
**Ethnoracial group**	741									
White/Canadian/European		492 (71.5)	43 (81.1)	0.051	Ref.					
Asian		112 (16.3)	2 (3.8)		0.26	(0.06, 1.05)	0.058			
Other		84 (12.2)	8 (15.1)		1.04	(0.49, 2.18)	0.928			
**Sexual orientation**	741									
Gay		587 (85.3)	43 (81.1)	0.056	Ref.					
Bisexual		29 (4.2)	6 (11.3)		2.07	(0.88, 4.87)	0.096			
Other		72 (10.5)	4 (7.5)		1.05	(0.37, 2.93)	0.933			
**Gender identity**	741									
Cisgender man		654 (95.1)	48 (90.6)	0.158	Ref.					
Transgender man and/or nonbinary		34 (4.9)	5 (9.4)		2.96	(1.16, 7.51)	**0.023**			
**Highest level of education**	741									
High school or less		77 (11.2)	12 (22.6)	**0.013**	Ref.					
Greater than high school		611 (88.8)	41 (77.4)		0.61	(0.32, 1.16)	0.130			
**Current employment**	741									
No		172 (25.0)	24 (45.3)	**0.001**	Ref.					
Yes		516 (75.0)	29 (54.7)		0.46	(0.26, 0.78)	**0.004**	Not selected		
**Currently have a regular primary health care provider**	741									
No		131 (19.0)	3 (5.7)	**0.015**	Ref.					
Yes		557 (81.0)	(94.3)		3.99	(1.24,12.77)	**0.020**			
**Unstably housed, P6M**	741									
Yes		33 (4.8)	15 (28.3)	**<0.001**	Ref.			Ref.		
No		655 (95.2)	38 (71.7)		0.21	(0.11, 0.39)	**<0.001**	0.40	(0.20, 0.82)	**0.013**
**Self-reported HIV status**	741									
HIV negative/unknown		529 (76.9)	22 (41.5)	**<0.001**	Ref.			Ref.		
Living with HIV		159 (23.1)	31 (58.5)		3.35	(1.93, 5.80)	**<0.001**	3.29	(1.72, 6.32)	**<0.001**
**HADS anxiety sub-score**	714									
Normal/mild		529 (79.4)	24 (50.0)	**<0.001**	Ref.			Ref.		
Moderate/severe		137 (20.6)	24 (50.0)		3.97	(2.25, 7.00)	**<0.001**	3.98	(2.17, 7.32)	**<0.001**
**HADS depression sub-score**	712									
Normal/Mild		631 (95.2)	41 (83.7)	**0.001**	Ref.					
Moderate/Severe		32 (4.8)	8 (16.3)		3.81	(1.80, 8.07)	**<0.001**	Not selected		
**P6M CAS with serodifferent partner**	733									
No		406 (59.7)	31 (58.5)	0.862	Ref.					
Yes		274 (40.3)	22 (41.5)		1.15	(0.67, 1.99)	0.609			
**P6M sex exchanged for drugs/money/goods**	738									
No		647 (94.3)	39 (75.0)	**<0.001**	Ref.					
Yes		39 (5.7)	13 (25.0)		4.26	(2.25, 8.07)	**<0.001**			
**P6M injection drug use**	741									
No		645 (93.8)	37 (69.8)	**<0.001**	Ref.					
Yes		43 (6.3)	16 (30.2)		4.90	(2.68, 8.96)	**<0.001**			
**Substance use disorder diagnosis (ever)**	729									
No		569 (83.8)	35 (70.0)	**0.012**	Ref.					
Yes		110 (16.2)	15 (30.0)		2.36	(1.29, 4.32)	**0.005**			
**P6M substance use**										
**Any sex drugs (crystal/GHB/erectile)**	735									
No		439 (64.4)	25 (47.2)	**0.012**	Ref.					
Yes		243 (35.6)	28 (52.8)		1.92	(1.12, 3.30)	**0.018**			
**Any club drugs (ecstasy/mushrooms/cocaine)**	734									
No		439 (64.4)	32 (61.5)	0.682	Ref.					
Yes		243 (35.6)	20 (38.5)		1.20	(0.69, 2.11)	0.519			
**Cannabis**	739									
No		302 (44.0)	20 (37.7)	0.374	Ref.					
Yes		384 (56.0)	33 (62.3)		1.32	(0.76, 2.29)	0.331			
**Cocaine**	738									
No		533 (77.8)	41 (77.4)	0.939	Ref.					
Yes		152 (22.2)	12 (22.6)		0.99	(0.52, 1.90)	0.980			
**Amphetamines (including Crystal meth)**	737									
No		482 (70.5)	27 (50.9)	**0.003**	Ref.					
Yes		202 (29.5)	26 (49.1)		2.17	(1.27, 3.72)	**0.005**	Not selected		
**Crystal meth**	736									
No		610 (89.3)	31 (58.5)	**<0.001**	Ref.					
Yes		73 (10.7)	22 (41.5)		4.57	(2.62, 7.96)	**<0.001**			
**Sedatives**	738									
No		563 (82.2)	36 (67.9)	**0.010**	Ref.					
Yes		122 (17.8)	17 (32.1)		2.16	(1.21, 3.86)	**0.009**	Not selected		
**Hallucinogens**	738									
No		523 (76.4)	39 (73.6)	0.649	Ref.					
Yes		162 (23.6)	14 (26.4)		1.51	(0.82, 2.78)	0.185			
**Opioids**	738									
No		652 (95.2)	42 (79.2)	**<0.001**	Ref.			Ref.		
Yes		33 (4.8)	11 (20.8)		4.21	(2.14, 8.27)	**<0.001**	2.38	(1.09, 5.20)	**0.029**

*Notes*. IRR: incidence rate ratio; aIRR: adjusted incidence rate ratio; CI : confidence interval; Ref.: reference group; HADS: Hospital Anxiety and Depression Scale; P6M: past six months; CAS: condomless anal sex; Not selected: variables that were initially entered into the multivariable model but were subsequently removed during model refinement based on minimization of the Quasi-likelihood Information Criterion (QIC) and assessment of type III tests of fixed effects. Variables without this designation (and without corresponding multivariable statistics) were not selected as candidate variables for the multivariable model and were therefore not entered due to pre-selection constraints related to the limited number of psychosis events. This analysis excludes the 57 participants who had a psychosis diagnosis during the wipeout period.

* Psychosis diagnosis from the interview date until the next visit or end of follow up.

Models used Poisson regression with generalized estimating equations and an autoregressive(1) correlation structure.

Significant *p*-values (<0.05) are bolded.

## Footnotes

1. GHB and GBL refer to gamma-hydroxybutyrate and gamma-butyrolactone, respectively.

2. Access to data provided by the Data Stewards is subject to approval but can be requested for research projects through the Data Stewards or their designated service providers. The following data sets were used in this study: COAST (Comparison of Outcomes and Service Utilization Trends), Consolidation File, MSP, and DAD. You can find further information regarding these data sets by visiting the PopData project webpage. https://my.popdata.bc.ca/project_listings/18-223. All inferences, opinions, and conclusions drawn in this publication are those of the authors, and do not reflect the opinions or policies of the Data Stewards.

3. Local Health Areas (LHAs) are the smallest geographic health-service delivery units defined by the British Columbia Ministry of Health, and there are 163 LHAs currently covering the entire province (Government of British Columbia & Ministry of Health, 2022). See the “Health Boundary Maps” portal for a full list of LHA boundaries (Government of British Columbia & Ministry of Health, 2025).
