# Supplementary material for: Substance Use and Incidence of Psychosis Diagnoses Among Gay, Bisexual, and Other Men Who Have Sex with Men Compared with Males from the General Population: A Matched Cohort Study from Metro Vancouver, Canada
Source: Subst Use Misuse. Author manuscript; Available in PMC 2026 Aug 4. (PMC13435960; doi:10.1080/10826084.2026.2682299)
Supplement: Supp 1 [file NIHMS2189863-supplement-Supp_1.docx]

**Substance Use and Incidence of Psychosis Diagnoses among Gay, Bisexual, and Other Men Who Have Sex With Men Compared with Males From the General Population: a Matched Cohort Study From Metro Vancouver, Canada**

*Supplement*

**Table S1**

*Characteristics of Momentum GBM Included vs. Excluded from COAST Administrative Data Linkage*

| **Variable** | **Total N** | **Excluded  (N=344)** | | **Included  (N=798)** | | **p-value** |
| --- | --- | --- | --- | --- | --- | --- |
|  |  | **N** | **(%)** | **N** | **(%)** |  |
| **Age (categorical)** | 1142 |  |  |  |  |  |
| Less than 30 |  | 195 | (56.7) | 245 | (30.7) | <0.001 |
| 30 to 44 |  | 104 | (30.2) | 295 | (37.0) |  |
| 45 or more |  | 45 | (13.1) | 258 | (32.3) |  |
| **Ethnicity** | 1142 |  |  |  |  |  |
| White/Canadian/European |  | 204 | (59.3) | 583 | (73.1) | <0.001 |
| Asian |  | 62 | (18.0) | 116 | (14.5) |  |
| Other |  | 78 | (22.7) | 99 | (12.4) |  |
| **Sexual orientation** | 1142 |  |  |  |  |  |
| Gay |  | 284 | (82.6) | 698 | (87.5) | 0.089 |
| Bisexual |  | 29 | (8.4) | 47 | (5.9) |  |
| Other |  | 31 | (9.0) | 53 | (6.6) |  |
| **Unstable housing** | 1134 |  |  |  |  |  |
| Yes |  | 38 | (11.0) | 68 | (8.6) | 0.195 |
| No |  | 306 | (89.0) | 722 | (91.4) |  |
| **Currently employed** | 1142 |  |  |  |  |  |
| No |  | 87 | (25.3) | 222 | (27.8) | 0.377 |
| Yes |  | 257 | (74.7) | 576 | (72.2) |  |
| **Self-reported HIV status** | 1142 |  |  |  |  |  |
| HIV Negative/Unknown |  | 302 | (87.8) | 587 | (73.6) | <0.001 |
| Living with HIV |  | 42 | (12.2) | 211 | (26.4) |  |
| **HADS Anxiety Sub-Score** | 1115 |  |  |  |  |  |
| Normal/Mild |  | 231 | (70.4) | 588 | (74.7) | 0.140 |
| Moderate/Severe |  | 97 | (29.6) | 199 | (25.3) |  |
| **HADS Depression Sub-Score** | 1115 |  |  |  |  |  |
| Normal/Mild |  | 312 | (94.8) | 744 | (94.7) | 0.905 |
| Moderate/Severe |  | 17 | (5.2) | 42 | (5.3) |  |
| **P6M exchanged sex for drugs/money/goods** | 1135 |  |  |  |  |  |
| No |  | 321 | (94.1) | 719 | (90.6) | 0.046 |
| Yes |  | 20 | (5.9) | 75 | (9.4) |  |
| **P6M injection drug use** | 1142 |  |  |  |  |  |
| No |  | 334 | (97.1) | 730 | (91.5) | 0.001 |
| Yes |  | 10 | (2.9) | 68 | (8.5) |  |
| **Self-Reported doctor-diagnosed substance use disorder diagnosis (ever)** | 1118 |  |  |  |  |  |
| No |  | 305 | (89.7) | 660 | (84.8) | 0.029 |
| Yes |  | 35 | (10.3) | 118 | (15.2) |  |
| **P6M substance use** |  |  |  |  |  |  |
| **Opioids** | 1138 |  |  |  |  |  |
| No |  | 326 | (95.0) | 721 | (90.7) | 0.013 |
| Yes |  | 17 | (5.0) | 74 | (9.3) |  |
| **Amphetamines** | 1140 |  |  |  |  |  |
| No |  | 250 | (72.7) | 504 | (63.3) | 0.002 |
| Yes |  | 94 | (27.3) | 292 | (36.7) |  |
| **Crystal meth** | 1139 |  |  |  |  |  |
| No |  | 308 | (89.5) | 655 | (82.4) | 0.002 |
| Yes |  | 36 | (10.5) | 140 | (17.6) |  |

*Note.* P6M refers to “past 6 months.”

**Table S2**

*Frequency of ICD-9 and ICD-10 Codes Used in the Psychosis Case Definition*

| **Codes** | **Diagnostic description** | **Momentum GBM (n = 110)** | | | **COAST Matches (n = 240)** | | | |  |
| --- | --- | --- | --- | --- | --- | --- | --- | --- | --- |
| ***ICD-9 codes for physician visits (MSP)*** | | **All Codes n (%)** | **First Code n (%)** | | **All Codes n (%)** | | **First Code n (%)** | |  |
| 291 | Alcohol-induced psychoses | 12 (10.9%) | 6 (5.5%) | | 54 (22.5%) | | 30 (12.5%) | |  |
| 292 | Drug-induced psychoses | 72 (65.5%) | 46 (41.8%) | | 114 (47.5%) | | 65 (27.1%) | |  |
| 295 | Schizophrenic disorders | 54 (49.1%) | 25 (22.7%) | | 145 (60.4%) | | 95 (39.6%) | |  |
| 297 | Delusional disorders | 16 (14.5%) | 7 (6.4%) | | 47 (19.6%) | | 14 (5.8%) | |  |
| 298 | Other nonorganic psychoses | 42 (38.2%) | 24 (21.8%) | | 110 (45.8%) | | 28 (11.7%) | |  |
| ***ICD-10 codes for hospitalizations (DAD)*** | |  | |  | |  | |  | |
| F20 | Schizophrenia | 2 (1.8%) | 0 (0.0%) | | 40 (16.7%) | | 4 (1.7%) | |  |
| F22 | Persistent delusional disorders | 0 (0.0%) | 0 (0.0%) | | 5 (2.1%) | | 1 (0.4%) | |  |
| F23 | Brief psychotic disorder | 1 (0.9%) | 0 (0.0%) | | 3 (1.3%) | | 1 (0.4%) | |  |
| F28 | Other nonorganic psychotic disorders | 0 (0.0%) | 0 (0.0%) | | 1 (0.4%) | | 0 (0.0%) | |  |
| F29 | Unspecified nonorganic psychosis | 11 (10.0%) | 2 (1.8%) | | 32 (13.3%) | | 9 (3.8%) | |  |

*Notes.*

1. “All codes” reflects the number of individuals for whom the diagnostic code was applied at any point during the lookback window or follow-up; individuals may appear under multiple codes.
2. “First code” reflects the earliest applied code, which determined incident psychosis diagnosis date.
3. Totals for "first code" for COAST matches exceed 240 because seven individuals received more than one code on the same day (e.g., concurrent MSP and DAD coding).
